# Models using private general practitioners to provide caesarean deliveries at five South African district public hospitals: insights for public-private contracting for obstetric care in rural areas

**DOI:** 10.1080/16549716.2023.2241811

**Published:** 2023-08-08

**Authors:** Geetesh Solanki, Emmanuelle Daviaud, Sue Fawcus, Vishal Brijlal, Tanya Doherty

**Affiliations:** a Health Systems Research Unit, South African Medical Research Council, Cape Town, South Africa; b Honorary Research Associate: Health Economic Unit, University of Cape Town, Cape Town, South Africa; c NMG Consultants and Actuaries, Cape Town, South Africa; d Department of Obstetrics and Gynaecology, University of Cape Town, Cape Town, South Africa; e Clinton Health Access Initiative, Pretoria, South Africa; f Department of Paediatrics and Child Health, University of Cape Town, Cape Town, South Africa

**Keywords:** Obstetric care, public-private partnership, Sub-Saharan Africa, national health insurance, universal health coverage

## Abstract

**Background:**

Harnessing of private sector resources could play an important role in efforts to promote universal access to safe obstetric care including caesarean delivery in low- and middle-income countries especially in rural contexts but any such attempt would need to ensure that the care provided is appropriate and patterns of inappropriate care, such as high caesarean delivery rates, are not reproduced for the entire population.

**Objective:**

To examine the contracting arrangements for using private general practitioners to provide caesarean delivery services in rural district hospitals in South Africa.

**Method:**

We utilised a mixed-method study design to examine the contracting models adopted by five rural district hospitals in the Western Cape, South Africa. Between April 2021 and March 2022, we collected routine data from delivery and theatre registers to capture the profile of deliveries and utilisation of contracted private GPs. We also conducted 23 semi-structured qualitative interviews with key stakeholders to explore perceptions of the contracting arrangements.

**Results:**

All five hospitals varied in the level of use of private general practitioners and the contracting models (three private in-sourcing models – via locum agencies, sessional contracts, and tender contracts) used to engage them. Qualitative interviews revealed insights related to the need for flexibility in the use of contractual models to meet local contextual needs, cost implications and administrative burden.

**Conclusion:**

Structured appropriately, private public partnerships can fill important gaps in human resources in rural district hospitals. Policy makers should look to developing a ‘contracting framework’ which requires compliance with a set of underlying principles but allows for flexibility in developing context specific contracting arrangements. These underlying principles should include a ‘risk’ based delivery model, adherence to public sector- evidence-based protocols, time-based rather than per delivery/type of delivery remuneration models, group liability arrangements, and processes to monitor outcomes.

## Background

Maternal and child health is a major public health concern in South Africa (SA). Whilst there has been progress in reducing maternal mortality from 160 per 100,000 live births in 2,000 to 119 per 100,000 live births in 2017 [1], there remain huge disparities between the public and private sectors in the distribution of obstetric care providers, models of care and outcomes [2]. In line with patterns seen in other countries, the case fatality rate (CFR) for caesarean delivery in the SA public sector is three times higher than for vaginal delivery, and 19% of the caesarean delivery-related CFR is associated with bleeding [2]. To address this, programmes to improve public sector skills and governance need to be implemented. These need to be complemented by the mobilisation of skills from the private sector given that the bulk of the resources lies in the private sector [3].

The mobilisation of private sector skills in the SA context would need to be in alignment with and complement the broader National Health Insurance (NHI) reforms taking place in the country towards achieving Universal Health Coverage (UHC). The NHI Bill [4] proposes that NHI will be a strategic purchaser of health care services from both the public and private sectors and for the entire SA population. As envisaged, the NHI will purchase services provided entirely by the public sector, it will purchase services directly from the private sector, and it will purchase services provided jointly by the public and private sectors through Public Private Engagements (PPE’s) which are seen as ”the deliberate, systematic collaboration of the government and the private health sector according to national health priorities, beyond individual interventions and programmes” [5] with the aim of harnessing private sector resources to further public health goals.

The proposed NHI will contract for obstetric services from both public and private providers and a key challenge for the NHI would be to develop contracting models that allow public and private resources to be mobilised to the benefit of the entire population. This is particularly important for rural district hospitals where there is a serious lack of skills and human resources to meet the service demands for obstetric care. However, attempts to harness the resources of the private sector to service the needs of the broader SA population would need to address the challenge of ensuring that the care provided is appropriate and patterns of inappropriate care, such as the high caesarean delivery rate (74%) of the private sector, for example [6], is not reproduced for the entire population. The development of contracting models that are responsive to these risks is critical for NHI to succeed in delivering UHC.

PPEs can take a variety of forms, and while there is a large empirical literature on PPEs in high-income countries, less is known about their operation in low- and middle-income countries (LMICs) [7,8], and understanding of the models that could be used in an NHI type environment is limited. NHI pilot site evaluations are the only research from SA to date on PPEs for clinical service provision, and these have only focused on the primary health care level [9–11].

Such exploration of different PPE models is also required to inform developments in other African countries in the process of implementing similar NHI financing arrangements. These include countries such as Ghana, Kenya and Tanzania [12]. We found no published research of experiences with PPEs for obstetric care in Africa or examples where GPs were contracted. The experience of different models of PPEs in LMICs more broadly is limited to specialist obstetricians and poorly documented. In India, public-private contracting for obstetric services utilises specialist obstetricians [13]. A public–private partnership programme in Gujarat state, India provides private obstetricians with a fixed sum per 100 births to provide care to eligible women below the poverty line. The remuneration model was specifically chosen as a disincentive to prevent unnecessary caesarean deliveries [14]. We found no studies where contracting arrangements were examined from a financial impact or health economics perspective.

Efforts to improve obstetric care are particularly important because caesarean delivery is a commonly performed surgical procedure and there is growing local concern about the appropriateness and safety of obstetric care in both the public and private sectors.

We carried out research to document the models used to contract private general practitioners (GPs) to provide caesarean delivery services in five rural district hospitals in the Western Cape [15], to qualitatively describe the experiences and perspectives of managers and doctors involved [16] and to examine the contracting arrangements that were used. The aim of this paper is to document our findings on the contracting arrangements that were used in the contracting of private GPs with a view to informing the development of public private arrangements for improving obstetric and maternal outcomes in rural areas in South Africa and LMICs more broadly.

## Methods

### Study design

We undertook operational research [17] with a mixed-method study design [18]. We chose a mixed-method study design incorporating both quantitative and qualitative data collections as this approach enabled us to document quantitatively the utilisation of contracted private GPs and to describe qualitatively the types of contracting models used and stakeholder perspectives of these models.

### Study setting

The setting for this research was the Western Cape Province where existing public-private contracting of GPs for caesarean delivery services was occurring due to human resource shortages in rural district hospitals. Five rural district hospitals within one rural district were chosen following engagement with provincial managers and obstetric clinical managers.

In South Africa, district hospitals provide level 1 (generalist) services to in-patients and outpatients (ideally on referral from a community health centre or clinic). District hospitals have between 30 and 200 beds, a 24-hour emergency service and an operating theatre. Generalists (medical officers) provide the services together with nursing staff and allied health professionals, some district hospitals have specialist family physicians serving as clinical managers but there are no obstetric or anaesthetic specialists at district hospital level [19]. Some district hospitals also have community service doctors. These are newly qualified doctors who have completed a two-year internship and are required to complete a further one year of community service [20].

Medical officers and GPs are both generalist doctors who have completed six years of medical school, two years of internship and a year of community service. At district hospital level, and in their GP practices; they are not restricted to working in one clinical discipline. In district hospitals, doctors may include community service doctors, medical officers who vary in level of expertise and family physicians who have a specialisation in Family Medicine (focused on being a district hospital doctor with public health as well as clinical knowledge and skills of all disciplines). All district hospital doctors are trained to perform emergency and elective caesarean delivery [19]. Of note, if caesarean delivery difficulty is anticipated, a more senior medical officer or family physician will perform it. Also, certain categories of caesarean delivery (emergency or elective) should not be done at district hospitals but referred to a regional hospital (e.g. BMI > 40, 2 or more previous caesarean deliveries, placenta praevia) [19]. GPs, whilst working in their own private practice as generalists (i.e. all clinical disciplines), may be contracted to a district hospital for specific functions, e.g. to perform surgery or anaesthesia for caesarean delivery. Depending on the timing of their contracted hours in the district hospital they may do emergency caesarean delivery or elective caesarean delivery (usually done during day time weekdays).

### Obstetric service model in the five district hospitals

All five district hospitals implemented the obstetric model of care practised in the public sector according to the National Department of Health Guidelines for Maternity care in SA [19]. Pregnant women received their antenatal care via a local primary health clinic unless triaged as high risk and referred for antenatal care in a hospital. They get admitted to the maternity ward of the local district hospital upon going into labour as none of the hospitals have primary care clinics in their catchment areas that perform vaginal deliveries other than in the case of emergencies and only during daytime hours (Table 1).

**Table 1.** Characteristics of participating hospitals*, contracting models and delivery profile.

Hospital**	(A)	(B)	(C)	(D)	(E)
**Hospital Characteristics:**					
Number of beds	35	50	75	85	118
Number of maternity beds (antenatal, labour and postnatal)	8	10	12	14	25
Number of clinics in the hospital catchment that perform vaginal deliveries	0	5 clinics that can do normal vaginal deliveries if referral to a district hospital is not possible, no 24- hour service	0	1 community day clinic, 4 clinics and 6 satellite services, none offer 24-hour service, can do normal vaginal deliveries if referral to a district hospital is not possible,	10 clinics that can do normal vaginal deliveries if referral to a district hospital is not possible, no 24- hour service
**Provider Profile:**					
Number of fulltime hospital employed doctors	0	6	6	10 (2 of which are specialist family physicians)	13
Number of community service doctors included in the total medical staff	0	4	2	4	5
Number of privately contracted GPs to assist with the obstetric service	One practice consisting of 5 GPs providing a 24- hour service undertaking both surgery and anaesthesia	2 GPs undertaking surgery	5 (2 for anaesthetics and 3 for surgery)	4 (2 surgical, 2 anaesthetic); 1 GP has a post-graduate diploma in anaesthetics, 1 GP has a post-graduate diploma in obstetrics, 1 GP is a specialist family physician	1 GP doing surgery only
Number of full-time equivalent private GPs (based on a public sector doctor contract of 40 hours per week plus 16 hours overtime)	0.9	0.3	2	0.9	0.2
**Contracting Model:**	Limited-bid tender entirely	Sessional contracts only	Sessional contracts only	Locum agency + Sessional contracts	Sessional contracts only
** Delivery Profile:**					
** Delivery type N (%)** **(1 April 2021–31 March 2022):**					
Vaginal (unassisted)	311 (75.5%)	356 (79.8%))	732 (73.9	1259 (85.6%)	1271 (73.6%)
Assisted vaginal (vacuum or forceps)	12 (2.9%)	9 (2.0%)	17 (1.7%)	13 (0.9%)	21 (1.2%)
** Caesarean deliveries**	**89 (21.6%)**	**81 (18.2%)**	**242 (24.4%)**	**199 (13.5%)**	**436 (25.2%)**
Total deliveries	412 (100.0%)	446 (100.0%)	991 (100.0%)	1471 (100.0%)	1728 (100.0%)
Elective caesarean deliveries	32 (36%)	26 (32%)	71 (29%)	56 (28%)	121 (28%)
Emergency caesarean deliveries	57 (64%)	55 (68%)	171 (71%)	143 (72%)	315 (72%)
** Private GP utilisation for Caesarean deliveries over a 12 month period (1 April 2021–31 March 2022):**					
** Private GP Surgeon**	89 (100.0%)	80 (98.8%)	94 (38.8%)	47 (23.6%)	42 (9.6%)
** Private GP Anaesthetist**	89 (100.0%)	0 (0.0%)	179 (74.0%)	51 (25.6%)	0 (0.0%)

*All hospitals were in the West Coast region of the Western Cape province of South Africa. Study period April 2021 to March 2022.

**To preserve the anonymity of the hospitals, they are given an alphabetical identifier.

The maternity ward is staffed by nurses and midwives who assess the risks of the expectant mother and based on public sector protocols make an assessment whether the birth should be via normal vaginal delivery or by caesarean delivery at the district hospital or whether the level of risk warrants referral to a regional hospital where more specialised expertise is available.

Normal vaginal deliveries occur in the delivery rooms of the district hospital by suitably qualified midwives/nurses, with doctors called upon only in the event where the birth becomes complicated for some reason. Assisted vaginal deliveries are performed by advanced midwives or medical officers. Cases earmarked for caesarean delivery, either emergency or elective, are booked for theatre with medical officers (public) or GPs (private) providing the surgical and anaesthetic services. Private GPs contracted to district hospitals are mainly utilised for theatre services either as a GP surgeon or GP anaesthetist to undertake caesarean deliveries or for gynaecological surgery including ectopic pregnancy, termination of pregnancy and uterine evacuation following spontaneous miscarriage. They may also be called for an assisted delivery if the establishment doctor is unable to manage a complicated delivery.

Decision making for caesarean delivery is made by the medical officers employed by the district hospital. Waiting time depends on the degree of urgency of the clinical condition and availability of theatre (district hospitals have one theatre for all surgical cases). High-risk cases may be referred to a regional hospital if time allows. Mostly, emergency obstetrics e.g. assisted vaginal delivery or managing post-partum haemorrhage is done by midwives and district hospital employed medical officers.

In terms of GPs involvement in decision making, this depends on the contracting model. In hospital A where one GP practice is contracted through a tender to provide services for the hospital and there are no government-employed doctors, then these private GPs make all obstetric care decisions (Table 1), whereas with the other two contracting models the on-site medical officers make the clinical decisions and then call out the private GP to provide surgical or anaesthetic services as required.

### Contracting model analytic framework

**Figure 1.**
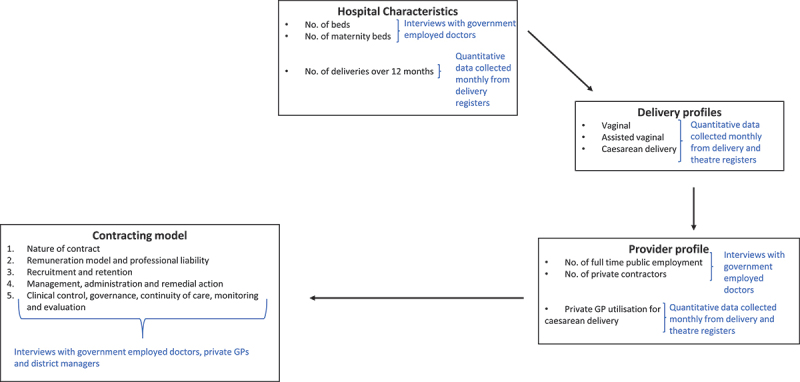
Analytic framework used to describe and analyse contracting models.

While all the hospitals relied on a ‘contracting in’ model (individual or agencies hired to provide services) to secure the services of the private GPs, a variety of contracting models were used. Drawing on previous work examining public-private partnerships for COVID-19 critical care [21], we developed an analytic framework to guide the collection and analysis of data required to describe the various contracting arrangements for obstetric care (Figure 1).

The analytic framework examined the public-private models used at the hospitals in terms of the characteristics of the hospital, the profile of deliveries over a 12-month period, the profile of obstetric care providers (publicly employed vs. contracted private GPs) and the contracting model(s) used to engage the private GPs. The contracting models were then further assessed using the following criteria: (1) nature of contract; (2) remuneration model and professional liability; (3) recruitment and retention; (4) management, administration and remedial action and (5) clinical control and governance, continuity of care, monitoring and evaluation.

#### Data collection and analysis

The analytic framework required the collection of quantitative and qualitative data.

##### Qualitative data collection

Data on the hospital characteristics, provider profile and the contracting model were collected via interviews for which we developed semi-structured interview guides, one for private providers (Supplementary file 1) and one for public providers (Supplementary file 2).

Interviews were undertaken by three of the investigators (TD, GS and ED) at the hospitals, practices of the private GPs and the district offices. The interviewers were two health economists and a health systems researcher with experience in qualitative interviewing. All interviews were conducted in a private consulting room or office and were undertaken in English. Interviews lasted between 30 and 60 minutes.

Interviews were undertaken with private GPs who had entered into public service contracts with each of the district hospitals to explore their perceptions of the contracting models. At each hospital, we also interviewed the public providers (doctors) to explore their perceptions of the public/private contracting models used. District managers responsible for service provision, human resources and finances were also interviewed to explore the specific contracting arrangements and financial implications.

Between April 2021 and March 2022 we undertook a total of 23 semi-structured qualitative interviews, including 3 district managers (2 male, 1 female), 12 private GPs (7 male, 5 female) and 8 government employed doctors (4 male, 4 female). All of the qualitative interviews were digitally recorded and transcribed.

##### Quantitative data collection

To document the profile of deliveries and utilisation of private GPs, we collected quantitative obstetric clinical data from the five district hospitals over a period of 12 months (1 April 2021–31 March 2022). Delivery profiles were captured from the delivery register and theatre register and included the following: mode of delivery and rates of caesarean delivery. For each caesarean delivery, we documented whether the surgeon and anaesthetist were a hospital employed medical officer or a private GP. Clinical data was collected by research nurses recruited for the purpose of the study. The monthly data was entered into a pre-set Excel spreadsheet.

### Qualitative data analysis

Transcripts were validated against the recordings to ensure accurate transcription. A framework analysis [22] approach was applied which is well suited to mixed-method research designs. We drew on our analytic framework to guide the analysis which was deductive in nature, focusing on perceptions of the three contracting models. The interviews were read and re-read by members of the team to familiarise ourselves with the content. Participant perceptions of the contracting models were mapped against the analytic framework. Five members of the research team (TD, ED, GS, SF and YB) read transcripts and met to agree on the mapping of qualitative findings to the framework. The interviewers had no prior interactions or relationships with any of the interviewees nor any pre-conceived biases about the benefits or pitfalls associated with these contracting arrangements. Reflexive discussions among the research team were held throughout the data collection process to reflect on emerging findings and to amend the interview guide to ensure that all aspects of the topic were explored.

### Quantitative data analysis

The quantitative data from hospital records was analysed in Excel using simple descriptive means and frequencies of annual deliveries, mode of delivery and caesarean delivery rates stratified by hospital. We describe the proportion of total annual caesarean deliveries at each hospital undertaken by private GPs as surgeons or anaesthetists.

The delivery profiles and utilisation of private GPs for caesarean deliveries are reported in Table 1. Qualitative results are reported using illustrative quotes and a description of the contracting models in Table 2.

**Table 2.** Key features of contracting models used in the five hospitals.

	Contracting Model			
	Full Time Public Employment	Private insourcing via Locum agency	Private in-sourcing on Sessional Basis	Private in-sourcing via tender
Nature of contract	Full time public employment. Period: Long term/permanent – terminated on resignation, retrenchment, retirement, disability/death Period: Long term/permanent – terminated on resignation, retrenchment, retirement, disability/death	Private GPs insourced via locum agency on ad-hoc basis to fill short-term ‘emergency’ gaps – mainly after hrs, nights, weekends Period: province has a 2 year convenience order with locum agencies. Agencies contract with GPs No benefits Period: province has a 2 year convenience order with locum agencies. Agencies contract with GPs No benefits	Private in-sourced to fill longer term ‘gaps’. Longer term (3 year) contracts with private GPs to cover fixed number of sessions (mainly after hours, weekends) per week/month Qualify for leave Longer term (3 year) contracts with private GPs to cover fixed number of sessions (mainly after hours, weekends) per week/month Qualify for leave	Full private in-sourced with Private GPs contracted to provide all clinical services for fixed per month fee basis Medium- term (3 year) contract No benefits Medium- term (3 year) contract No benefits
Remuneration model and professional liability	Salaried staff on full time employment contract. FTE: 40 hrs/week +16 hrs commuted overtime.Remunerated on Total Cost of Employment basis inclusive of (leave, sick leave, medical cover, pension). Overtime more expensive than using sessional doctorsCommuted over-time paid a third for standby. Medico-legal indemnity provided by the state FTE: 40 hrs/week +16 hrs commuted overtime. Remunerated on Total Cost of Employment basis inclusive of (leave, sick leave, medical cover, pension). Overtime more expensive than using sessional doctors Commuted over-time paid a third for standby. Medico-legal indemnity provided by the state	Time based remuneration – paid by call-out session fee for the GP and an intermediary fee to the agency. Day or night rates differ. Standby paid 1/3 of on site. Although locum per hour rates are higher, overall costs can be lower than contracting sessional staff. No leave or pay benefitsIndividual doctor to have personal medico-legal indemnity cover Individual doctor to have personal medico-legal indemnity cover	Time based remuneration – paid by session. Maximum 39hrs on site/month. Stand by 1/3. This ensures doctors paid on standby, even when low patient activity. Fixed monthly rate (same day or night), dependent on whether 5 years or 10 years’ experience. Leave included, no pension or medical aid. Hourly rates higher than permanently employed staff as ‘benefits’ not provided. Medico-legal indemnity provided by the state Fixed monthly rate (same day or night), dependent on whether 5 years or 10 years’ experience. Leave included, no pension or medical aid. Hourly rates higher than permanently employed staff as ‘benefits’ not provided. Medico-legal indemnity provided by the state	Fixed amount per month covering a ‘basket of services’ to be rendered. Annual increases incorporated as part of agreement (independent from public service wage increase)Doctors’ own medico-legal indemnity cover Annual increases incorporated as part of agreement (independent from public service wage increase) Doctors’ own medico-legal indemnity cover
Recruitment and retention	Recruitment depends on availability of posts and of candidates. Posts decided by provincial or national.Recruitment in rural areas difficult and turnover high. Posts decided by provincial or national. Recruitment in rural areas difficult and turnover high.	Recruitment not a challenge in areas close to cities.Locum agencies do not operate in more remote areas. Retention not an issue. Locum agencies do not operate in more remote areas. Retention not an issue.	Need to have posts. Posts decided by provincial or national. If sick/on leave, hospital must find replacement. Recruitment of sessional local private GPs currently not a challenge but concern as high number of rural private GPs retiring and unclear whether will be replaced. With local GPs, retention very high If sick/on leave, hospital must find replacement. Recruitment of sessional local private GPs currently not a challenge but concern as high number of rural private GPs retiring and unclear whether will be replaced. With local GPs, retention very high	Province advertises a tender to which interested parties can apply. Private Drs cover for own leave and sick leave. Minimum HR implications for hospitalRecruitment and retention concern of contracted party Private Drs cover for own leave and sick leave. Minimum HR implications for hospital Recruitment and retention concern of contracted party
Management, administration and remedial action	Easier to schedule for clinical rosters. Need to plan for gaps arising from staff turnover, leave etc. Abide by Department of Health Disciplinary process with verbal, written and final warnings. Well defined disciplinary processes which specify Remedial actions to be taken. Possibilities for Remedial action, and non-approval of probation. Corrective training with supervision. Dismissal possible for serious offences. Need to plan for gaps arising from staff turnover, leave etc. Abide by Department of Health Disciplinary process with verbal, written and final warnings. Well defined disciplinary processes which specify Remedial actions to be taken. Possibilities for Remedial action, and non-approval of probation. Corrective training with supervision. Dismissal possible for serious offences.	Least onerous administration and very responsive to changes in hospital needs. Monthly invoicing from agency. Remedial action relatively easy by indicating to agency that doctor no longer wanted. Remedial action relatively easy by indicating to agency that doctor no longer wanted.	Administratively like full time staff, sessions incorporated into rotations, paid monthly via payroll. etc. Remedial action: difficult during the contract but can be affected through non-renewal of contract. Remedial action: difficult during the contract but can be affected through non-renewal of contract.	Administratively onerous for set-up but minimum thereafter. Payment via monthly fixed amount.Remedial action: difficult in the course of the contract but can be affected through non-renewal of contract. Remedial action: difficult in the course of the contract but can be affected through non-renewal of contract.
Clinical control and governance, continuity of care, monitoring and evaluation.	Level of control: High Clinical decisions made by employed staff, required to adhere to treatment guidelines and protocols. Required to attend M&M and specialist meetingsHigh level of continuity of care – easier to get full time staff to work within the ‘team’ framework, abide by protocols, attend clinical governance sessions etc. Ongoing monitoring and evaluation processes in place though routine hospital data collection systems. Clinical decisions made by employed staff, required to adhere to treatment guidelines and protocols. Required to attend M&M and specialist meetings High level of continuity of care – easier to get full time staff to work within the ‘team’ framework, abide by protocols, attend clinical governance sessions etc. Ongoing monitoring and evaluation processes in place though routine hospital data collection systems.	Level of control: weak Although decisions re caesarean delivery made by employed staff, adherence to clinical protocols by locum doctors sometimes a problem as they come in on ad-hoc and short-term basis. Do not participate in M&M meetings, nor visits by specialist.No continuity of care. Uneven quality of staff. Monitoring is ad-hoc – via feedback from nurses, or doctors to the locum, sometimes review of records. Although decisions re caesarean delivery made by employed staff, adherence to clinical protocols by locum doctors sometimes a problem as they come in on ad-hoc and short-term basis. Do not participate in M&M meetings, nor visits by specialist. No continuity of care. Uneven quality of staff. Monitoring is ad-hoc – via feedback from nurses, or doctors to the locum, sometimes review of records.	Level of control: Relatively strong Decision re Caesarean delivery made by employed staff, sessional staff tend to adhere to protocols due to longer term contracts which allow performance to be monitored. Attendance of M&M and specialist outreach when possible dependent on timing of sessions (during/after hours). Continuity of care: Intermediate – develop relations with staff, but not patients. M&E via hospital records, feedback from nurses and staff. Decision re Caesarean delivery made by employed staff, sessional staff tend to adhere to protocols due to longer term contracts which allow performance to be monitored. Attendance of M&M and specialist outreach when possible dependent on timing of sessions (during/after hours). Continuity of care: Intermediate – develop relations with staff, but not patients. M&E via hospital records, feedback from nurses and staff.	Level of control relatively weak although practice may have a senior doctor who provides clinical governance. Clinical decisions ultimately made by private providers. Adherence to protocols high as contracts are longer term, accountability for services rests entirely with private provider and monitoring and evaluation systems are in place. Continuity of care: HighAttendance of M&M and specialist meetings dependent on time spent at hospital vs private practice). M&E via hospital records, feedback from nurses and staff. Clinical decisions ultimately made by private providers. Adherence to protocols high as contracts are longer term, accountability for services rests entirely with private provider and monitoring and evaluation systems are in place. Continuity of care: High Attendance of M&M and specialist meetings dependent on time spent at hospital vs private practice). M&E via hospital records, feedback from nurses and staff.

FTE = full-time equivalent; M&*M* = morbidity and mortality; M&E = monitoring and evaluation.

## Results

### Hospital characteristics, provider profile, contracting arrangements and delivery profile

The five district hospitals ranged in size from 35 beds (8 maternity) in hospital A to 118 beds (25 maternity) in hospital E. In hospital A, there are no government employed doctors, and the hospital is run entirely by a private GP practice consisting of five doctors who provide 24-hour cover to the hospital. The other four hospitals relied mainly on permanent publicly employed medical officers and/or community service medical officers but contracted the services of private GPs where needed. The number of contracted GPs ranged from 1 in hospital E to 5 in hospitals A and C but this translated into a maximum of 2 full time equivalent state-employed doctors in hospital C and 0.2 in hospital E (Table 1).

The hospitals secured the services of private GPs through a combination of three contracting models. These included limited-bid tender, fixed-term sessional contracts and use of locum agencies. Of the five hospitals, one used only the tender model, three used the sessional contract model and one used a combination of locum agency and sessional contracts (Table 1). All five hospitals had been contracting with private GPs using a combination of these contracting models for a minimum of 10 years prior to this research study.

Over the period 1 April 2021 to 31 March 2022, the total number of deliveries ranged from 412 in hospital A to 1728 in hospital E. The caesarean delivery rate ranged from 13.5% at hospital D to 25.2% at Hospital E. The utilisation of private GPs for surgery and anaesthesia for caesarean deliveries differed widely across the hospitals (Table 1).

### Characteristics of contracting models to meet human resource requirements of rural district hospitals

The full-time public employment and the three private-insourcing contracting models (via locum agency, sessional basis, tender) to meet human resource requirements for doctors in the obstetric service are characterised and summarised in Table 2.

### Public employment contract model

Medical officers in the public employment contract model are employed permanently, with full ‘benefits’ on a total cost of employment contractual basis. The hourly overtime and standby rates for full-time employees are higher than for in-sourced private GPs. The state covers medico-legal indemnity for permanently employed doctors and sessional contract private GPs. Recruitment of permanent staff is dependent on availability of posts (decision on posts is the remit of the provincial and national departments) and attracting doctors to work in rural areas is challenging with high turnover rates. The administrative and management burden is lighter with this contracting model as the posts are permanent, the doctors can be easily scheduled in the rotations and are paid via the general staff payroll system. The full-time employment model allows for a high level of clinical control, governance continuity of care and monitoring and evaluation through adherence to the provincial clinical guidelines, norms and standards, compulsory continuing education, attendance at audit meetings and annual performance review.

#### Private GP in-sourcing models

##### Nature of contract

For the three private insourcing models, the level of in-sourcing is lowest in the locum agency model, highest in the tender model and somewhere in between in the sessional basis model. Contracts are short for the locum model, determined monthly as required, and both sessional and tender contracts are renewable every three years.

##### Remuneration model and professional liability

Remuneration for locum and sessional contracts is on a ‘time’ basis. The tender contract is based on a fixed monthly fee for providing an agreed-to set of services as specified in the tender document. Interviewees described that the remuneration rates for locums were agreed between the hospital and the locum agencies. The agency then agrees on the hourly remuneration rate with the private GPs.

What we generally do is, we just send our needs out to all the locum agencies and then they quote and we generally take the cheapest one or if we deviate from that, you must motivate as to why. (Government employed doctor, Hospital D)

We normally just pay the agency. What the agency pay the doctor, we don’t know. (District finance manager)

For sessions, the rates were set by the province with GP’s deciding whether they were willing to provide services at the rates offered. The rates for tender contracts were negotiated as a district finance manager explained:

Because the tender is purely negotiated rates. There is a ballpark of a rate per hour plus a bit of rate per kilometer and you come up with a figure. But if they tender for this and it’s slightly above that, the question is, are you going to accept it, or are you not? Is it more expensive than trying to appoint permanent? So it becomes a business transaction purely based on what they can offer with what skill and how permanent that service provider is. (District finance manager)

A private GP also shared that with a tender contract they receive no benefits such as maternity leave, which they have to factor into their bid.

There’s no, we’re talking about benefits. There are no maternity benefits, there’s none of that sort of thing. We have to sort that out amongst ourselves. (Private GP, hospital A)

Interviewed respondents perceived the sessional contracts to be cheaper for the hospital, especially for after-hours cover, than employing more permanent staff on the establishment as one government doctor described:

I just want you to be here on, Tuesday, which is my elective caesarian section list and you can with your DA (Diploma in Anesthetics) do all the patients very safely and then, the rest I’ll just use as home time (on stand by). Versus employing her as a full-time kind of medical officer. It will be cheaper, especially for the afterhours stuff because you can stretch the sessions quite far. It’s a balancing act. (Government employed doctor, hospital D)

In terms of medico-legal indemnity, the State provides cover for those on sessional contracts but locums and those providing services via tender are required to have their own cover which needs to be factored into their tender budget as a district human resource manager explained:

The medico legal also because if they’ve got a sessional appointment by us, then it’s fine. Then they don’t have to worry about medico legal costs. So there’s a lot of stuff that they must think of before they put their bid in and that’s what they’re actually bidding for. (District HR manager)

##### Recruitment and retention

While recruitment via locum agencies was relatively easy, a district HR manager shared that the locum model of private insourcing is only feasible for hospitals close to urban centres:

The closer you are to the metro, the more readily available the agency doctors. So (name of hospital) is fairly close to (name of suburb). That’s fine for a doctor to quickly run. But on the other side of the mountain, it’s more difficult. (District HR manager)

Recruitment and retention for the sessional model was easier than with full-time employment, while the recruitment and retention issues were a concern for the party awarded the tender in the tender model.

##### Management, administration and remedial action

The nature and level of administrative and management burden varies, with private in-sourcing via locum agencies carrying the least burden due to the contract being with the agency and not the individual doctors. The sessional basis model requires processes for renewal/issuing of the contracts every three years when compared to the permanent public employment model. A hospital employed doctor shared that with sessional contracts the hospitals still need to find replacements if the contracted sessional doctor is sick whilst with locum agencies the agency has to find a replacement if the allocated doctor is unavailable.

The sessional comes with its own drawbacks because they are basically staff members and they’re entitled to leave and if they’re sick it’s not their problem it’s your problem. Whereas a locum agency, the doctors sick it’s the agency’s problem to provide somebody else with the same set of skills. (Government employed doctor, hospital D)

The tender model has a high set-up burden in terms of drafting the tender specifications, advertising, screening applications and processing the contract but is then relatively easy to manage.

In terms of remedial action in the event that a contract needs to be terminated, this can be quickly taken with the locum model since the hospital can use a different agency. With the sessional and tender models, remedial action is difficult in the course of the contract, although feedback on hospital adverse events and training can be provided. Non-renewal of the contract would be the method for termination of the sessional or tendered service.

Where remedial action is required, there are opportunities for on-site corrective counselling and training input. There is also an established Department of Health progressive disciplinary process to respond to different degrees of poor performance by an individual health care worker. This can lead to dismissal for serious offences but this can be a difficult prolonged process, and dismissals may be difficult to effect, should they be necessary.

##### Clinical control and governance, continuity of care, monitoring and evaluation

The level of clinical control and governance, continuity of care, training of staff, and capacity to monitor and evaluate the services being provided was highest with the permanent employment model, least with the locum model and somewhere in between for sessional and the tender models as an HR manager described:

It’s not ideal because you don’t have control, unlike the outsourced limited bid tender service. You don’t have control over a locum as you would like. It’s not long term. It’s really not ideal. (District HR manager)

Since contracted GPs often cover after-hours or night shifts, it is difficult to incorporate them into continuing education sessions or audit meetings. In the locum and sessional contract models, some clinical governance is created through the medical officers making clinical decisions regarding the need for a caesarean delivery.

##### Choice of contracting model

District managers have brought up the need for flexibility in the choice of contractual models to meet local contextual needs, particularly the availability of suitably qualified doctors in the town.

I think what the short answer is what’s available in that geographic area in that town will dictate the kind of contract. (District HR manager)

We don’t have enough medical officers to cover our service. So we’ve now put in an extra community service doctor into obstetrics. I’m expecting that locum expenditure to fall away. I think we also use it sort of flexibly, so if somebody’s on maternity leave, you can potentially take some of the savings on their overtime expenditure that we can then allocate towards locum expenditure to cover it there. (District director)

## Discussion

Low- and middle-income countries including SA face considerable challenges in ensuring access to safe and appropriate obstetric care for rural districts, especially access to caesarean delivery [2]. This study, although limited in terms of the number of hospitals included, has documented several different contracting models that are used by rural district hospitals to meet the human resource requirements of obstetric services. The variation in local contexts necessitated the use of different contracting models, and hospital managers emphasised the need for flexibility in the choice of contracting models to meet local contextual needs, rather than specifying a ‘one size fits all’ contracting model. Stakeholders shared that contracting through locum agencies is only feasible for district hospitals close to urban centres and the limited bid tender model requires the presence of a GP practice that can be contracted as a single contracting unit. Decision making on which contracting model to use requires district managers to consider the financial costs of contracting-in versus employing more permanent doctors.

The five hospitals varied in the level of private GP use and the contracting models used to engage them. The caesarean delivery rate across the study hospitals ranged from 13.5% to 25.2% - in line with caesarean delivery rates reported at public health facilities in South Africa and well below the 75% caesarean delivery rate reported for the private sector in SA [6]. The caesarean delivery rates across the hospitals were not related to the level of use of the private GPs or the contracting models used. A common feature of all three contracting models is a time-based rather than per delivery/type of delivery-based remuneration model. The time taken for caesarean deliveries is generally less and more predictable than for normal vaginal deliveries. There is therefore an in-built incentive to carry out caesarean delivery with a remuneration model based on delivery type. With the time-based remuneration, this incentive is removed as the GP is remunerated for a set number of hours per shift or month, not on the number or type of delivery and this arguably could have contributed to the low C section rate. The relatively low caesarean delivery rate at these five hospitals is most likely related to the case severity load, since complicated caesarean deliveries are not done at district hospitals [19].

This research did not explore the requirements of public sector regional or tertiary hospitals in urban areas where PPEs might include in-sourcing of private obstetric and anaesthetic specialists or out-sourcing of patients to private facilities when the public hospital is overloaded. This type of PPE might have more problematic issues than described in this paper in terms of optimising caesarean delivery rates which are known to be over 74% among private specialists [6], and because of the very different models of maternity care between public and private hospitals. Further research to seek opinions from relevant stakeholders would be important so that models of care and PPE arrangements for these urban contexts can be suggested.

With the current acute shortage of health care workers in low- and middle-income countries [23] and major imbalances between public and private sectors, it has never been more urgent to assess how different human resource strategies might be used to improve population-based health outcomes. This is particularly important for rural areas where there are generally no or very few obstetric and anaesthetic specialists and models using GPs need to be considered.

Globally, government purchasing health care services from the private sector has been proposed as a strategy to address the inequities in access to healthcare. Proponents of the approach point to potential benefits, such as increased provider competition, better quality of care, increased utilisation and equity, improved health outcomes, efficiency and transparency, while opponents advocate for strengthening publicly delivered services as they deem the private sector to be contributing to the inequitable provision of services. However, public-private partnerships in poorly regulated health systems with limited public governance capacity can have unintended consequences [8,24]. Insights from research on the consequences of PPEs in obstetric care in LMICs are limited, with most of the research having been undertaken in India and involving contracting with specialist obstetricians [13,25]. In some states, private providers are contracted into public facilities to increase skills and human resources while in other states, costs for services are subsidised within private facilities [15]. A facility survey and interviews with providers in Maharashtra state found that the contracting of specialists did not greatly increase emergency obstetric care service outputs at facilities, except in facilities with determined leadership. Contracting in specialists was useful for non-emergency conditions, but not for obstetric emergencies. The contracts were poorly monitored and inadequate infrastructure, longer distance to private specialists, insufficient financial provision for contracting in, and poor management capacities were barriers to effective implementation of contracting in [26].

This paper is the first to our knowledge to describe contracting models using private GPs to improve access to quality obstetric care in rural districts. The small sample size and lack of economic analysis limits the ability to draw definitive conclusions, particularly around the costs of the different contracting models relative to increased public sector doctor employment. However, there are several important considerations in the use of contracted providers for obstetric care that this research has highlighted that may assist in ensuring a high quality of care under a situation of greater public private engagement: (1) the adoption of a ‘risk’ based delivery model, with a staff nurse/midwife performing the routine deliveries, with doctors managing complicated cases, performing surgical obstetric procedures with referral to higher level of care when required (2) tight clinical control and governance processes adhering to public sector protocols (3) time based rather than per delivery/type of delivery-based remuneration models, (4) group rather than individual professional liability arrangements (5) processes to monitor outcomes, evaluate performance and apply remedial measures where required. Initiatives to develop PPEs with GPs in rural district hospitals, whether in the current, transition or fully implemented NHI environment, should consider incorporating these features as guiding principles.

In the current pilot stage of NHI implementation only primary care GPs have been contracted to provide services in public sector clinics, and these pilots have used the sessional contract model. There have been no pilot evaluations of hospital level contracting of private providers. The limited-bid tender model provides the opportunity for both in- and out-sourcing and could be considered for contracting units, such as GP practices or private hospital groups under NHI.

### Limitations

The sample size of five hospitals is small and as a result, the generalisability of the findings is limited. We were unable to access financial information related to the contractual arrangements and were therefore unable to assess the costs and relative costs of the different contracting models, and we were not able to find any other comparable studies to draw upon.

## Conclusion

Obstetric care is a major public health concern in South Africa and provides an ideal ‘case study’ for examining the potential health systems challenges for the proposed NHI. The findings of this study indicate that PPEs involving private GPs can support the human resource requirements for obstetric care in rural district hospitals. Based on the findings of this research, we recommend that for PPEs in rural areas, rather than prescribing a standardised ‘one size fits all’ contracting model, policymakers should be looking to develop a 'contracting framework’ which requires compliance with a set of underlying principles but allows for flexibility in developing context specific contracting arrangements. Developing, piloting, and implementing public-private models are necessary for responsiveness to the goal of UHC, and can be used to improve obstetric care, while broader health system reforms are taking place. We also recommend further research in the area, particularly around the financing arrangements and implications of the various contracting models.

## Supplementary Material

Supplemental Material

## Paper context

Harnessing private sector resources could play an important role in promoting safe obstetric care in rural contexts but any such attempt would have to ensure that appropriate care is appropriate. Policymakers should develop a ‘contracting framework’ which requires compliance with underlying principles but allows for flexibility in developing context specific contracting arrangements. The principles include a ‘risk’ based delivery model, adherence to public sector protocols, time-based remuneration models, group liability arrangements, and outcome monitoring.

## References

[1] World Health Organisation. Trends in maternal mortality 2000-2017. Geneva: World Health Organisation; 2019.

[2] National Department of Health. Saving mothers 2017-2019. Seventh report on confidential enquiries into maternal deaths in South Africa. Pretoria: National Department of Health; 2020.

[3] Barber SL, Kumar A, Roubal T, Colombo F, Lorenzoni L. Harnessing the private health sector by using prices as a policy instrument: lessons learned from South Africa. Health Policy. 2018 May;122:558–13. doi: 10.1016/j.healthpol.2018.03.018 29622381

[4] Bland RM, Rollins NC, Solarsh G, Van den Broeck J, Coovadia HM. Maternal recall of exclusive breast feeding duration. Arch Dischildhood. 2003 Sep;88:778–783. doi: 10.1136/adc.88.9.778 PMC1719625 12937095

[5] Whyle EB, Olivier J. Models of public–private engagement for health services delivery and financing in Southern Africa: a systematic review. Health Policy Plann. 2016;31:1515–1529. doi: 10.1093/heapol/czw075 27296061

[6] Solanki G, Fawcus S, Daviaud E, Doherty T. A cross sectional analytic study of modes of delivery and caesarean section rates in a private health insured South African population. PloS One. 2019;14:e0219020. doi: 10.1371/journal.pone.0219020 31247013 PMC6597103

[7] Hellowell M. Are public–private partnerships the future of healthcare delivery in sub-Saharan Africa? Lessons from Lesotho. BMJ Global Health. 2019;4:e001217. doi: 10.1136/bmjgh-2018-001217 PMC6509596 31139440

[8] African Development Bank. Developing coordinated public-private partnerships and systems for financing health in Africa: experiences from Africa and India. Abidjan: African Development Bank; 2017.

[9] Genesis Analytics. Evaluation of phase 1 implementation of interventions in the National Health Insurance (NHI) pilot districts in South Africa. Johannesburg: Genesis Analytics; 2019.

[10] Hongoro C, Funani IIN, Chitha W, Godlimpi L. An assessment of private general practitioners contracting for public health services delivery in O.R. Tambo District, South Africa. J Public Health Afr. 2015;6:525–525. doi: 10.4081/jphia.2015.525 28299145 PMC5349272

[11] Mureithi L, Burnett JM, Bertscher A, English R. Emergence of three general practitioner contracting-in models in South Africa: a qualitative multi-case study. Int J Equity Health. 2018 Oct 5;17:107. 30286772 10.1186/s12939-018-0830-0 PMC6172712

[12] Cashin C, Dossou J-P. Can national health insurance pave the way to universal health coverage in Sub-Saharan Africa? Health Syst Reform. 2021 Jan 1;7:e2006122. doi: 10.1080/23288604.2021.2006122 34965364

[13] Iyer V, Sidney K, Mehta R, Mavalankar D. Availability and provision of emergency obstetric care under a public–private partnership in three districts of Gujarat, India: lessons for universal health coverage. BMJ Global Health. 2016;1:e000019. doi: 10.1136/bmjgh-2015-000019 PMC5321320 28588914

[14] Salazar M, Vora K, Sidney Annerstedt K, De Costa A. Caesarean sections in the in the context of the Chiranjeevi Yojana public private partnership program to promote institutional birth in Gujarat, India; does the embedded disincentive for caesarean section work? Int J Equity Health. 2019;18:17–17. doi: 10.1186/s12939-019-0922-5 30678731 PMC6345034

[15] Doherty T, Solanki G, Daviaud E, Bartmann Y, Hawkridge A, Fawcus S. Utilisation of private general practitioners to provide caesarean deliveries in five rural district public hospitals in South Africa: a mixed-methods study. BMJ Open. 2023;13:e067663. doi: 10.1136/bmjopen-2022-067663 PMC9980375 36858464

[16] Doherty T, Fawcus S, Daviaud E, Bartmann Y, Solanki G. Experiences of public-private contracting for caesarean delivery in rural district public hospitals: A qualitative interview study. PLOS Glob Public Health. 2023;3:e0001335. doi: 10.1371/journal.pgph.0001335 37155593 PMC10166521

[17] Remme JHF, Adam T, Becerra-Posada F, D’Arcangues C, Devlin M, Gardner C, et al. Defining research to improve health systems. PLOS Med. 2010;7:e1001000. doi: 10.1371/journal.pmed.1001000 21124816 PMC2993153

[18] Creswell John W, Plano Clark Vicki L. Choosing a mixed methods design. Designing and conducting mixed methods research. 2nd ed. Los Angeles, London, New Dehli: Sage; 2011. p. 53–106.

[19] South African National Department of Health. Guidelines for maternity care in South Africa: A manual for clinics, community health centres and district hospitals. 4th ed. Pretoria: National Department of Health; 2016. p. 1–146.

[20] Department of Health. A district hospital service package for South Africa. Pretoria: Department of Health; 2002.

[21] Solanki G, Blecher M, Cornell J, Crisp N, Engelbrecht B, Manning M, et al. COVID-19: insights from contracting the private sector for critical care. In: Govender K, George G, and Padarath A, et al., editors. South African health review 2021. Durban: Health Systems Trust; 2021. p. 83–93.

[22] Gale NK, Heath G, Cameron E, Rashid S, Redwood S. Using the framework method for the analysis of qualitative data in multi-disciplinary health research. BMC Med Res Methodol. 2013 Sep 18;13:117. doi: 10.1186/1471-2288-13-117 24047204 PMC3848812

[23] Ahmat A, Okoroafor SC, Kazanga I, Asamani JA, Millogo JJS, Illou MMA, et al. The health workforce status in the WHO African region: findings of a cross-sectional study. BMJ Glob Health. 2022 May;7:e008317. 10.1136/bmjgh-2021-008317 PMC9109011 35675966

[24] Hort K, Nachtnebel M, O’Mahoney A, Pillai N. Purchasing arrangements with the private sector to provide primary health care in underserved areas (Policy brief, Vol. 3 No. 1 2014). Manila: Asia Pacific Observatory on Health Systems and Policies; 2014.

[25] Chaturvedi S, Randive B. Public private partnerships for emergency obstetric care: lessons from Maharashtra [original article]. Indian J Community Med. 2011 Jan 1;36:21–26. 21687376 10.4103/0970-0218.80788 PMC3104703

[26] Randive B, Chaturvedi S, Mistry N. Contracting in specialists for emergency obstetric care- does it work in rural India? BMC Health Serv Res. 2012 Dec 31;12:485. 23276148 10.1186/1472-6963-12-485 PMC3572412

