# Supplementary material for: Models using private general practitioners to provide caesarean deliveries at five South African district public hospitals: insights for public-private contracting for obstetric care in rural areas
Source: Glob Health Action. 2023 Aug 8;16(1):2241811. doi: 10.1080/16549716.2023.2241811 (PMC10411302; doi:10.1080/16549716.2023.2241811)
Supplement: Supplemental Material [file ZGHA_A_2241811_SM0662.zip › Supplementary_file_1_topic_guide_pr_6.docx]

**Public-private contracting models for obstetric services in the Western Cape**

# **STUDY TOPIC GUIDE:** Private providers

Interview data: ________

Name of Hospital that the person is contract to:___________________________

Name of interviewee:_____________________ Designation:________________________________

**Before the interview:**

1. Introduce myself to interviewee, brief explanation of study topic, study purpose “We would like to talk to you about your experience of providing maternity care and *(for hospitals with existing contracts)* within government hospitals
2. Read Information Sheet, reassure confidentiality, signing of consent form
3. State expected length of interview -45min
4. Ask permission to record interview

**Use the below suggested questions but probe further and be flexible. Let the interviewee direct the conversation.**

**Topic Guide:**

Please tell me a little about your experience as a doctor:

- - How long they have worked in private practice
  - Main role and responsibilities (specialist, GP, obstetric or anaesthetic focus)

For private specialists: Questions related to their private hospital work

Can you please describe the model of care in your private hospital setting (E.g. role of midwives vs obstetricians)

What is your caesarean rate in your private practice; do you think it is too high or too low?

Can women themselves decide to have a CS on request?

Do you think medico-legal concerns influence your decision making?

Have you had personal experiences of medico-legal claims in your career? If yes. What was your experience of these

Does your private hospital have a system of auditing maternal and perinatal outcomes?

Does the hospital ever identify adverse complication from C sections; or cases where an adverse outcome occurred because the C section was not done or done too late? Are these cases discussed? Any action taken?

Questions related to their public-private contracting

When did you first enter into a contract with government?

What kind of contract do you have currently? (probe sessions, fee for service etc)

What has been your experience of working in government hospitals?

What services are you contracted to provide?

How are you indemnified against medico-legal claims when you work in a public health facility? Probe MPS or government insures?

Tell me about the kinds of cases that you are called to manage (probe: complicated NVDs, complicated C/S, elective C/S etc)

Are there differences in the decision-making and clinical management between your private practice setting and the public maternity unit setting? Tell me about them.

Does the communication and team dynamics differ in the public maternity setting? How?

What are the advantages for you of entering into a contract with a government hospital?

What are the challenges of working in a government hospital? How can these be overcome?

How do you feel about the contract you currently have (probe: fairness of remuneration)

Would it be beneficial for you to have greater involvement in the public sector? In what way?

End

- Reiterate confidentially “I just want to remind you again that everything we have discussed here will remain between me, the research team on this study and yourself. No outside person will have access to this information, and your name will not be on any of the publications and reports that will be written about this study.”

Thank you....
