# Supplementary material for: Models using private general practitioners to provide caesarean deliveries at five South African district public hospitals: insights for public-private contracting for obstetric care in rural areas
Source: Glob Health Action. 2023 Aug 8;16(1):2241811. doi: 10.1080/16549716.2023.2241811 (PMC10411302; doi:10.1080/16549716.2023.2241811)
Supplement: Supplemental Material [file ZGHA_A_2241811_SM0662.zip › Supplementary_file_2_Topic_guide_go_7.docx]

**Public-private contracting models for obstetric services in the Western Cape**

# **STUDY TOPIC GUIDE:** Government doctors

Interview data: __________________________ Name of Hospital:___________________________

Name of interviewee:_____________________ Designation:________________________________

**Before the interview:**

1. Introduce myself to interviewee, brief explanation of study topic, study purpose “We would like to talk to you about your experience of providing maternity care and *(for hospitals with existing contracts)* especially your interaction with private providers
2. Read Information Sheet, reassure confidentiality, signing of consent form
3. State expected length of interview -45min
4. Ask permission to record interview

**Use the below suggested questions but probe further and be flexible. Let the interviewee direct the conversation.**

**Topic Guide:**

Please tell me a little about your role in this hospital:

- - How long they have worked there
  - Main role and responsibilities
  - Do they only work in government or also do some RWOPs in the private sector

Can you tell us a bit about how maternity care works in your hospital e.g., staffing, do midwives or doctors do normal deliveries, when do doctors get involved in labour ward care?

What is your primary need to improve the provision of maternity care in this hospital?

Do doctors or nurses in your hospital have the skills to perform assisted vaginal deliveries e.g. Forceps and Ventouse?

Who makes the decision to perform CS?

Can women themselves decide to have a CS on request?

What is your caesarean rate in this hospital; and do you think it is too high or too low?

Do you think medico-legal concerns influence decision making?

Have you had any experiences of medico-legal claims related to obstetric care?

Are you aware of any obstetric medico-legal claims currently or in the past at this hospital?

Does your hospital have a system of auditing maternal and perinatal outcomes? If yes, probe how often they meet, who is part of the committee, actions taken in response to adverse outcomes

Does the hospital ever identify adverse complications from C sections; or cases where an adverse outcome occurred because the C section was not done or done too late?

What level of provider performs the surgery and who provides anaesthesia?

Does your hospital have scheduled and time tabled (i.e. sessions) provision of service from private providers, or only occasionally (i.e. in emergency situation). If yes, probe when this started, what prompted this arrangement, what needs does it address?

*If hospital has currently contracted private providers:*

Can you describe who the private providers are (specialist obstetricians, anaesthetists or GPs?)

What services are the private providers contracted to provide and when are they called upon?

What experiences have you had of working with private providers? Tell me about the decision-making process to call on a private provider (probe: short staff, inexperienced staff, complicated delivery etc)

Do the communication and team dynamics differ when a private provider is leading the care for a public patient.

*For both hospitals that do and those that don’t currently have private providers:*

In your opinion are there differences in the decision-making and clinical management of labour and delivery between government and private providers? Can you tell me about them.

What are the advantages of bringing private providers into government hospitals?

What are the challenges of bringing private providers into government hospitals? How can these be overcome?

Would it be beneficial for your maternity unit to have greater involvement of private providers? In what way? How could the public and private sectors work together more to support each other?

Are there any circumstances when there might be benefit in sending some of your patients to a private facility (eg lack of beds or theatre space, or lack of skill)?

End

- Reiterate confidentially “I just want to remind you again that everything we have discussed here will remain between me, the research team on this study and yourself. No outside person will have access to this information, and your name will not be on any of the publications and reports that will be written about this study.”

Thank you....
